# Recognizing and controlling airborne transmission of SARS‐CoV‐2 in indoor environments

**DOI:** 10.1111/ina.12697

**Published:** 2020-06-19

**Authors:** Joseph G. Allen, Linsey C. Marr

**Affiliations:** ^1^ Healthy Buildings Program Department of Environmental Health Harvard T.H. Chan School of Public Health Boston MA USA; ^2^ Department of Civil and Environmental Engineering Virginia Tech Blacksburg VA USA

Sharing indoor space has been confirmed as a major risk factor in transmission of SARS‐CoV‐2. A study of over 7000 cases found that all outbreaks involving three or more people occurred indoors.^1^ Thus, identifying the dominant modes of transmission is an urgent public health priority so that appropriate control strategies can be selected and deployed. Here, we present three lines of evidence supporting the potential for airborne transmission and recommend steps to mitigate the risk in indoor environments.

## 1 DETECTION AND SURVIVAL OF CORONAVIRUS IN AIR

SARS‐COV‐2 viral RNA in air has been detected in several studies in hospitals, including at distances greater than 2 m from patients and in outdoor air in crowded areas near a hospital and a department store.^2^, ^3^, ^4^ One study found that the majority of these viruses was associated with microscopic droplets (ie, aerosols) of diameter 2.5 microns and smaller,^3^ which can remain suspended in air for 2 hours or more. Another detected SARS‐COV‐2 in aerosols in the size range of 1‐4 microns.^2^ Furthermore, viral RNA has been detected on the surfaces of an air exhaust outlet and fan, places where direct transfer from an infected person could not occur.^5^ While these studies assayed viral RNA, the finding that SARS‐COV‐2 in fine aerosols (<5 microns) has a half‐life of 1 hour in terms of infectivity raises the possibility that some airborne virus is infectious.^6^ Concluding that transmission of SARS‐COV‐2 can occur via large droplet spray requires an assumption that the virus survives in such droplets. It is also reasonable, then, to assume the same for virus survival in aerosols. This is supported by empirical evidence. Previous studies have shown that other viruses survive equally well, if not better, in suspended aerosols compared to large droplets on surfaces.^7^, ^8^, ^9^

## 2 ASYMPTOMATIC INFECTIVITY

There are reports of asymptomatic transmission of SARS‐CoV‐2.^10^ By definition, asymptomatic patients are not coughing or sneezing, which means they are not frequently generating large droplets. Therefore, for these asymptomatic patients, other modes of transmission, namely fomite and airborne, must be occurring. A study on patients with confirmed influenza infection has shown that infectious virus in aerosols smaller than 5 microns can be released by regular breathing and talking, without coughing.^11^ This is of concern because high shedding of infectious SARS‐CoV‐2 in the throat has been reported in individuals with no or mild symptoms .^12^

## 3 AEROSOL PHYSICS

While the traditional distinction between “droplet” and “airborne” transmission of infectious disease has been useful for setting guidelines on the use of personal protective equipment, it has also established a false dichotomy in understanding the behavior of viruses in the air. Virus‐containing droplets that are released by breathing, talking, and coughing span a continuum of sizes, from 0.01 to hundreds of microns. It is impossible for someone to release “large droplets” (>5 microns) without also releasing smaller ones.^13^ Thus, transmission that is purported to occur via the spray of large droplets could in fact be occurring through inhalation of much smaller droplets at close range. In fact, a physics‐based simulation suggests that the majority of exposure at close range occurs by inhalation of small droplets rather than by contact with large droplets that land on the mouth, nose, and eyes, unless the people are closer than 30 cm or the droplets are very large.^14^

## 4 RECOMMENDATIONS

Evidence is emerging indicating that, in addition to transmission via large droplets and fomites, SARS‐CoV‐2 is also transmitted via inhalation of aerosols. Recognition of this transmission route is critically important because there are measures we can take to reduce the risk of airborne transmission. These include

- increasing outdoor air ventilation rates above current minimums
- using high‐efficiency filtration for recirculated air (MERV 13 or greater)
- verifying that sensitive areas, such as bathrooms and rooms where infected patients are cared for in hospitals and senior homes, are negatively pressurized relative to adjacent areas
- managing air flow direction and speed to prevent spread of aerosols across occupants
- consideration of additional technological controls, such as UV germicidal irradiation and portable air purification, in areas and situations where typical building‐level controls are not sufficient
- using N95 respirators in healthcare settings.

Last, these ventilation‐focused engineering controls must be supported by strategies that address fomite transmission because viruses in droplets and aerosols that have settled on the floor and other surfaces can be resuspended in air, where they can lead to inhalation exposure to the virus. Cleaning surfaces using vacuums with HEPA filtration and frequently cleaning and disinfecting surfaces are strategies that may also help reduce secondary airborne transmission.

## AUTHOR CONTRIBUTION

Joseph Allen: Conceptualization (lead); Investigation (equal); Writing‐original draft (equal); Writing‐review & editing (equal). Linsey Marr: Conceptualization (supporting); Investigation (equal); Writing‐original draft (equal); Writing‐review & editing (equal).
